# Transcription Factor Promiscuity Drives Regulatory Rewiring and Evolvability in Gene Networks in Bacteria

**DOI:** 10.1002/advs.202520406

**Published:** 2026-01-23

**Authors:** Tiffany B. Taylor, Alan M. Rice

**Affiliations:** ^1^ Department of Life Sciences University of Bath Bath UK; ^2^ UCD National Virus Reference Laboratory University College Dublin Dublin Ireland

**Keywords:** crosstalk, experimental evolution, gene regulatory network, *Pseudomonas fluorescens*

## Abstract

This special issue marking the University of Bath's 60th anniversary offers an opportunity to reflect on nearly a decade of research into the evolution of gene regulatory networks (GRNs) from members of the  lab and elsewhere. Our goal is to understand how GRNs rewire and how new transcription factor (TF) functions evolve. Using an experimental evolution model system with the soil bacterium *Pseudomonas fluorescens*, we have been able to observe TF rewiring in real time, providing unique insights into the principles of GRN evolution. In this perspective, we highlight three central discoveries from this system: a hierarchical pattern of TF rewiring, in which some regulators act as preferred “first responders”; the critical influence of expression level and mutational accessibility on whether a TF can be recruited for novel function; and the role of crosstalk (non‐cognate binding) as the raw material for adaptive innovation. Together, these findings reveal why evolutionary pathways are often constrained and thus strikingly repeatable. By identifying what makes a TF evolvable, we are beginning to predict, and potentially direct, evolutionary outcomes. Finally, we consider open questions and emerging technologies that have the potential to transform our understanding of GRN rewiring and its relationship with evolvability.

## Graphical abstract

When a master transcription factor (TF) is lost, bacteria can rapidly rewire gene regulatory networks by co‐opting related regulators. Using experimental evolution in *Pseudomonas fluorescens*, we show that TF promiscuity (low‐level, non‐cognate binding) provides the raw material for rewiring. Successful co‐option follows a predictable hierarchy governed by expression level, activation potential, and pre‐existing affinity. Crosstalk, often dismissed as noise, is a latent source of regulatory variation that selection can amplify into adaptive innovation. Graphical abstract by Melisa Morales and Miranta Kouvari from Science Graphic Design.

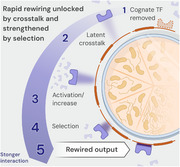

## 1 Introduction

Understanding how new transcription factor (TF) functions arise and how gene regulatory networks (GRNs) rewire during evolution remains poorly understood. The biophysical properties of TF‐DNA interaction must balance high specificity with the inevitability of some off‐target binding, known as crosstalk. While crosstalk often imposes fitness costs via the misregulation of off‐target genes, its persistence suggests a nuanced balance between cost and benefit. Crosstalk may expose latent connections within networks, predisposing them to rapid adaptation and genetic innovation. However, its persistence cannot be attributed to future utility—evolution lacks foresight. Instead, persistence may arise because: natural selection is too inefficient to refine interactions beyond “good enough,” [1]; perfect binding specificity is constrained by biochemistry; or, we have mischaracterized crosstalk, overlooking a functional role. Each explanation highlights gaps in our fundamental understanding.

Across the tree of life, bursts of transcriptional novelty are repeatedly associated with major evolutionary transitions. For example, the emergence of new morphological structures or the diversification of developmental programs [2]. Comparative genomics shows that paralogous TF families expanded by gene duplication provide a latent reservoir for functional innovation (redundant duplicates can diverge to evolve new roles) [3]. Predicting which paralog will be co‐opted, however, is not possible with our current understanding. Even deeply conserved regulatory architectures can be repurposed by evolution. For example, the entire enhancer landscape controlling cloacal development in ancestral vertebrates was co‐opted to drive digit formation in tetrapods [4], illustrating how novel traits are often achieved by redeploying pre‐existing network modules in new contexts. To help address these gaps in our current understanding, empirical evidence is required.

Experimental evolution with bacteria offers a powerful model system for studying GRN rewiring due to its genetic tractability and short generation times [5]. In our approach, we engineer network configurations to favor rewiring, with an interest in understanding its adaptive consequences and mechanistic underpinnings. Our experimental system uses *Pseudomonas fluorescens* SBW25 [6], a versatile soil bacterium with a large genome and an abundance of regulatory proteins [7]. We deleted the master regulator gene *fleQ*, which normally controls expression of all flagellum genes, rendering the bacterium non‐motile [8]. Motility is an ideal target phenotype because the regulatory architecture of the flagellar system is well characterized, the trait is readily observable, and within the background of our genetic construct, it can only be restored through GRN rewiring. Moreover, selection is easily imposed by modulating nutrient availability or medium viscosity. On soft‐agar plates, cells cannot spread outward to new nutrients unless they regain flagellar function, imposing strong selection on motility recovery. Within as little as 96 h, populations consistently regained flagella‐driven motility via a reproducible two‐step evolutionary pathway [9]. With this approach, we aim to provide empirical data on long‐standing theoretical questions about how GRNs balance robustness with adaptability. Crucially, results from this approach show that principles typically invoked to explain macroevolutionary innovation (TF co‐option, network modularity, and TF promiscuity) are visible, testable, and mechanistically explicable in the microevolutionary experiments we conduct in the lab, positioning this system as a uniquely integrative model for understanding the mechanisms of regulatory network evolution, by bridging the gap between short‐term adaptation and the long‐term emergence of novel gene network architectures.

*P. fluorescens* SBW25 encodes a family of 22 structurally related TFs known as σ^54^/RpoN‐dependent enhancer‐binding proteins (RpoN‐EBPs) [10], including FleQ itself and many other regulators with similar domain architecture. This large family provides multiple candidate regulators potentially capable of rescuing flagellar function in FleQ's absence [11, 12]. Such built‐in homology creates a rich landscape to explore questions of TF innovation: Which TFs can take over a lost regulatory function, and why some but not others? The *P. fluorescens* model enables us to address these questions experimentally by tracking evolution across replicate populations and genetic backgrounds. The phenotype of interest—motility on soft agar—is easy to screen, and whole‐genome sequencing of evolved isolates pinpoints the causal mutations underlying flagellar rescue. Moreover, *P. fluorescens* thrives in the laboratory and tolerates genetic manipulation, allowing us to create specific mutant backgrounds (e.g., multiple TF knockouts or controlled TF expression strains) to test hypotheses related to GRN rewiring.

This model system has uncovered general principles of GRN evolution that extend beyond our model species (Box 1). Our findings show that the capacity for crosstalk between diverged TFs confers regulatory networks with exceptional robustness [13, 14]. They further suggest that some degree of evolutionary resilience is an unavoidable outcome of regulatory systems shaped by gene duplication [15, 16]. Functional convergence of this kind has likely occurred many times in evolutionary history, and our experimental approach essentially retraces, in reverse, the same gene duplication and divergence processes that underpin adaptive diversity in all gene families [17, 18].

## 2 Hierarchical Rewiring of Transcription Factors

Across diverse systems, evolutionary studies increasingly reveal that TFs are not equally likely to be co‐opted, raising the question of what determines which regulators become accessible evolutionary “entry points” for network rewiring [e.g., 19, 20]. To understand why certain TFs are repeatedly co‐opted while others are excluded, it is essential to consider the architecture and regulatory context of each candidate. In our system, FleQ is the master regulator of flagellar gene expression, sitting at the top of a hierarchical network that coordinates the expression of genes responsible for flagellum biosynthesis and motility [21]. Deletion of *fleQ* disables this regulatory module entirely, rendering cells non‐motile. Our initial studies revealed that when *fleQ* was deleted, mutations that rescued flagellar motility were exclusively within the Ntr system, co‐opting NtrC to replace FleQ's lost function [22]. NtrC is part of the same protein family (RpoN‐EBPs) as FleQ and normally activates genes for nitrogen uptake and assimilation via phosphorylation from its sensor kinase two‐component partner, NtrB [23]. RpoN‐EBPs are TFs that bind to RpoN (also known as σ^54^) to activate transcription of target genes. Bacteria typically have a single RpoN protein but multiple different EBPs, which allows a single RpoN to regulate different functions by interacting with various EBPs [24].

Under strong selection for motility, *P. fluorescens* SBW25∆*fleQ* follows a consistent two‐step evolutionary path: first, mutations that boost NtrC activity appear, allowing NtrC to weakly activate flagellar genes; and second, a “switch‐of‐function” mutation refines NtrC's role toward FleQ target sites and away from NtrC target sites [8]. This two‐step rewiring via NtrC occurred in every replicate population, to the exclusion of all other potential rewiring routes [20]. To test whether this repeatability reflected absolute constraint, we constructed a double knockout strain (Δ*fleQ* Δ*ntrC*) and again applied selection for motility rescue on soft agar. The bacteria still evolved to restore flagella production, but this time by recruiting a different TF. In the absence of NtrC, a putative regulator called PFLU1132 was rewired to rescue flagellar motility [13]. PFLU1132 is an RpoN‐EBP encoded in the same operon as the sensor kinase PFLU1131, forming a two‐component system. Notably, PFLU1132 is a FleQ homolog, and it had not previously been implicated in flagellar regulation [10].

In the Δ*fleQ* Δ*ntrC* background, all motile isolates acquired mutations PFLU1131 as the first step, likely hyperactivating PFLU1132. Second‐step mutations were distributed across the genome and varied between lines, but all increased expression of the rewired TF, PFLU1132. This revealed a hierarchy in TF accessibility: NtrC is the preferred route to flagellar rewiring, and PFLU1132 rewires only when NtrC is unavailable [13]. In a triple mutant (∆*fleQ* ∆*ntrC* ∆*PFLU1132*), no motile isolates evolved across many replicate lines within a 6‐week period. This suggests that beyond NtrC and PFLU1132, other paralogous regulators in *P. fluorescens* are highly unlikely to innovate FleQ function under the tested conditions. The discovery of the NtrC‐PFLU1132 hierarchy has important implications. It suggests that, under the experimental conditions used to select for motility recovery, evolutionary routes to rewiring are uneven, likely driven by context‐dependent mutational opportunity, evolutionary history, and/or regulatory affinity [25, 26].

## 3 Mutational Accessibility of Expression Levels Shapes Transcription Factor Evolvability

Why were NtrC and PFLU1132 the only TFs to successfully rewire the flagellar network in our experiments? And, why was NtrC the preferred route? TF expression has been identified as a key gatekeeper of regulatory innovation, with only those regulators that can readily achieve high activity states becoming viable substrates for rewiring [27, 28, 29]. Transcriptomic data from our previous study suggested that low expression levels constrain the ability of TFs to rewire, because the diverse mutations we observed that rescued FleQ function via rewiring of PFLU1132 were all associated with increased expression [13]. In general, most TFs are expressed only weakly or under specific conditions, likely because higher expression carries fitness costs through misregulation [30]. NtrC is unique within its TF family by virtue of the GRN architecture it sits within: a single mutation in its cognate kinase (*ntrB*) caused constitutive phosphorylation of NtrC. However, because NtrC can also activate its own promoter through a positive feedback loop, this same mutation also rapidly amplified NtrC‐P levels in the cell [22]. The resulting flood of active NtrC increased the probability of binding to non‐cognate promoters, including those normally controlled by FleQ, thereby weakly reactivating the flagellar regulon.

The importance of TF abundance became even clearer when we artificially increased the expression of other RpoN‐EBPs. In a follow‐up study, we placed alternative RpoN‐EBP family members under inducible promoters to test whether they could also rescue motility when expressed above native levels under selection for motility [14]. We uncovered six additional TFs within the same RpoN‐EBP protein family (FleR, HbcR, GcsR, DctD, AauR, and PFLU2209) that, when expressed at high levels, yielded previously unseen mutations enabling motility rescue.

High expression of a TF can compensate for weaker intrinsic activity or affinity [31]. With more molecules of the active TF present, even low‐probability binding events to target promoters become more likely, following simple mass‐action principles [32]. In our previous experiments, NtrC and PFLU1132 were more accessible to evolution as high expression and/or activation could be achieved via a few mutations. For NtrC, a single nucleotide change conferred both high activity and high expression. For PFLU1132, a single in‐frame deletion in its sensor kinase PFLU1131 increased phosphorylation, and subsequent mutations increased its expression [13]. We hypothesized that other regulators likely required more complex or less probable mutational routes to achieve comparable increases in expression. When we proactively boosted expression, however, several alternative rewiring pathways were revealed [14]. This underscores a general principle: the ease with which a TF's expression can be increased (or its activity elevated in a given environment) strongly influences its evolutionary potential to rewire.

**FIGURE 1:**
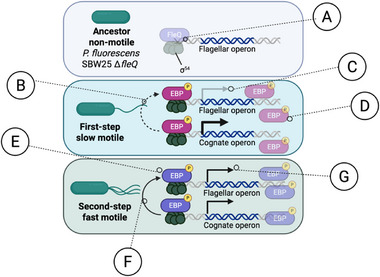
Stepwise evolution of transcription factor rewiring restores motility in *Pseudomonas fluorescens* SBW25 Δ*fleQ*. Deletion of the master flagellar regulator *fleQ* abolishes expression of flagellar genes (A), resulting in a non‐motile ancestor. Under selection for motility, populations consistently evolved through a reproducible two‐step rewiring pathway. In the first step (B–D), low‐level crosstalk allows a non‐cognate enhancer‐binding protein (EBP) to weakly bind unoccupied FleQ target sites (B), producing weak expression of the flagellar operon (C). A single activating mutation in the EBP's cognate kinase (D) causes hyperphosphorylation and overactivation, increasing both its own expression and that of its native regulon. In the second step (E–G), additional mutations refine the EBP–DNA interaction, strengthening non‐cognate binding to FleQ promoter sites (E, F). This refinement increases expression of the flagellar operon while reducing expression of the EBP's original target operon, reflecting a shift in binding affinity from cognate to non‐cognate sites (G). Together, these steps illustrate how crosstalk, activation potential, and expression level interact to enable transcription factor rewiring and functional innovation in bacterial gene regulatory networks. Created in BioRender; https://BioRender.com/322pm6x.

In our recent work, we summarized that three key properties facilitate TF innovation: (1) the ability to achieve high activity (e.g., via mutation in cognate histidine‐kinase that increases activation), (2) the ability to attain high expression levels, and (3) some preexisting low‐level affinity (Figure 1) [13]. When these conditions are met, a TF becomes a prime candidate for evolutionary rewiring. NtrC and PFLU1132 satisfied all three in our flagellar rescue model, whereas most other paralogs did not, until we intervened experimentally to satisfy property 2 (high expression) [14].

## 4 Crosstalk as a Driver of TF Innovation

Promiscuous, non‐cognate binding is now understood to be an unavoidable consequence of TF biophysics and paralog diversification, making low‐level crosstalk a fundamental feature of regulatory networks and potentially exploitable by natural selection [26, 33, 34]. While often dismissed as noise, these low‐level interactions can provide the raw material to natural selection for new regulatory functions to evolve [35, 36, 37, 38, 39]. Non‐specific binding between TFs and DNA arises from both biophysical limits on interaction fidelity [32, 40] and the shared ancestry of TF families, which retain overlapping specificities after duplication and divergence [17, 41]. Crosstalk in locally adapted individuals is costly, as it disrupts the precision and efficiency of gene regulation, and cells have evolved mechanisms to insulate pathways and mitigate their effects [30, 42]. Yet its persistence, and the variability in extent across lineages [43], raises fundamental questions: are there conditions or evolutionary scenarios where crosstalk is, in fact, advantageous?

Evolutionary benefits of crosstalk may include linking different signaling pathways to coordinate growth, metabolism, and adaptation, especially in environments where stimuli occur in sequence [24]. This capacity to integrate signals may allow rapid responses to fluctuating conditions or stress [44]. In our system, transcriptomic data showed that deleting *fleQ* corresponded with reduced expression of nitrogen assimilation‐associated genes, suggesting some pre‐existing crosstalk between the networks [9]. The interaction is assumed to be neutral and a product of relatedness between diverged protein families. However, once selection for motility was imposed, crosstalk provided variation that could be amplified and refined by selection. This mechanism also explains why completely unrelated regulators (with different DNA‐binding specificities) did not rescue motility: they lacked even weak affinity for FleQ binding sites under native expression levels. By contrast, the structural similarity of NtrC and PFLU1132 to FleQ conferred a basal affinity for flagellar gene promoters, which natural selection could reinforce by amplifying mutations that increased affinity toward novel target sites or probability of non‐cognate interactions [45].

**FIGURE 2:**
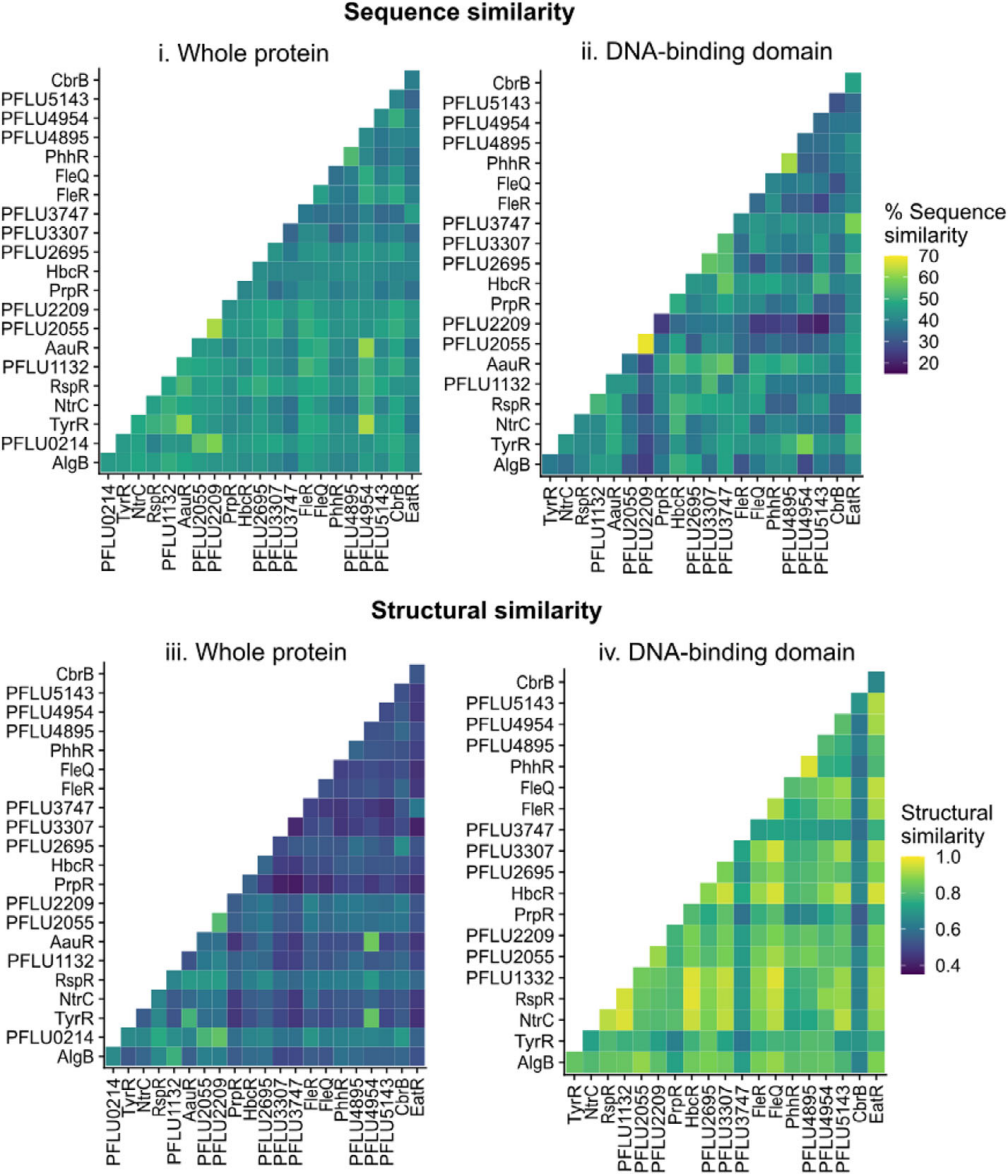
Pairwise similarity comparisons across the *P. fluorescens* σ^5^ ^4^–enhancer‐binding protein (EBP) family. The heatmap depicts a range of similarity scores with high scores represented in yellow and low scores in dark blue. For each EBP pair, full‐length sequence similarity (i) and DNA‐binding domain (DBD) sequence similarity (ii) were calculated using SIAS, and predicted structural similarity (TM‐scores) derived from AlphaFold models and evaluated with TM‐Align for full‐length proteins (iii) and the DBD (iv) [86]. PFLU0214 is excluded from the DBD comparisons due to an incomplete DBD annotation, and AauR is excluded from structural DBD comparisons due to short pairwise alignments in this region (<25 amino acids). The figure shows for sequence and structure, at the global and DBD‐specific levels, each reveals different patterns of similarity, but no single metric reliably forecasts regulatory rewiring potential on its own.

However, structural homology did not explain the order in which EBPs were recruited to compensate for lost FleQ function [10]. This is likely because global structural similarity across the entire protein is unlikely to be the main determinant of regulatory co‐option. Members of the RpoN‐EBP family share a highly conserved central catalytic domain, flanked by a more rapidly evolving N‐terminal regulatory domain and C‐terminal DNA‐binding domain (DBD) [46, 47]. Because the DBD directly determines promoter recognition, we might expect DBD similarity to be a strong predictor of promoter co‐option. To test this, Figure 2 compares (i and iii) whole‐protein sequence and structural similarity, and (ii and iv) DBD‐region sequence and structural similarity across the protein family. None of these measures relative to FleQ are notably elevated for NtrC or PFLU1132, despite their repeated and predictable recruitment during motility‐recovery evolution. Instead, our experiments suggest that successful functional rescue requires only weak non‐cognate promoter interactions, while the identity and timing of the recruited regulator are better explained by TF expression level and the mutational accessibility of promoter sites under selection. Thus, although DBD similarity can highlight regions of potential promoter compatibility, recruitment into a new regulatory role is strongly context‐dependent, rather than being determined by sequence or structural similarity alone.

Cells typically evolve mechanisms to suppress crosstalk because misexpression is costly [35]. Regulatory networks are optimized for specificity; for example, negative selection and feedback loops minimize unwanted interactions between paralogous two‐component systems in bacteria [30, 48, 49, 50, 51]. However, under novel conditions or loss of a master regulator, non‐cognate interactions create variation that natural selection can exploit [52]. This principle has been observed elsewhere. An elegant experiment with bacteriophage repressors showed that the λ repressor (λ cI) is inherently more evolvable than the related phage P22 repressor (c2) because λ cI is more robust to mutations and can tolerate binding to alternate DNA sites [11]. In effect, λ cI had more latent flexibility (promiscuity) in DNA recognition, enabling it to acquire new regulatory capabilities more readily.

The role of crosstalk in our system illustrates a broader principle: just as promiscuous enzyme activities can pave the way for new metabolic functions to evolve [53], promiscuous DNA‐binding by TFs can enable new regulatory networks to emerge [54]. These results remain consistent with evolution as bricolage—tinkering with what is at hand—but show that tinkering occurs within a constrained and biased landscape, making some rewiring outcomes repeatable and predictable [55].

## 5 Open Questions and Future Directions

While we have made progress in understanding bacterial GRN rewiring, many exciting questions remain. Here, we highlight several promising directions for future research.

A key question in GRN rewiring is how TFs innovate when their ancestral role remains important for survival. In our evolution experiments, the repeated recruitment of NtrC to replace lost FleQ function occurred under conditions where nitrogen assimilation was not strongly selected. In natural environments where nitrogen sensing is essential (which includes nitrogen‐limited soils, rhizospheres, or host‐associated niches), this co‐option could impose regulatory conflict, particularly through intermediate promoters recognized by both native and non‐cognate TFs. One proposed evolutionary resolution is gene duplication followed by divergence to reduce crosstalk [17], a mechanism broadly supported as a route to regulatory complexity but not one that can yet be predicted from sequence or structure alone. Massively parallel reporter assays have shown that dual‐TF promoter recognition can either accelerate or impede adaptation, depending on the cost of crosstalk [45]. Crucially, this question is no longer only theoretical, but is experimentally accessible, offering a promising direction for future work to empirically quantify duplication‐driven specialization and crosstalk costs in rewiring networks.

Advances in artificial intelligence and machine learning offer powerful ways to predict and design regulatory interactions. Deep learning models trained on DNA sequences and TF binding data can predict potential binding sites, including cryptic sites not detectable under normal conditions [56, 57, 58]. Integrating such models with evolutionary studies could be transformative. By predicting networks of weak interactions for each TF, combined with knowledge of GRN structure, we may anticipate which non‐cognate targets are most accessible to innovation through a few mutational steps. AI tools may also simulate the effect of specific mutations on TF binding [59], specificity, or activity [60], allowing virtual exploration of evolutionary pathways [61].

Our experimental evolution studies typically observe outcomes at the population level (e.g., appearance of motile mutants after several days). However, new single‐cell techniques now make it possible to observe the dynamics of network evolution in unprecedented detail. Fluorescent reporter strains [45], microfluidics [62], and time‐lapse imaging [63] could reveal whether subsets of cells transiently overexpress key TFs before mutations arise. Single‐cell RNA sequencing (which is only recently optimized for bacterial cells) [64] could capture gene expression heterogeneity in evolving populations, identifying “primed” expression states that predispose cells to adaptive trajectories [65].

High‐throughput assays are also revolutionizing how we study crosstalk. Massively parallel reporter systems now test thousands of TF–DNA interactions simultaneously; for example, a recent study applied this approach to a TetR‐family repressor, profiling ∼18,000 candidate binding sequences and uncovering hundreds of latent sites beyond the wild‐type operator [45]. In parallel, DAP‐seq (DNA affinity purification sequencing) and the high‐sensitivity PADIT‐seq (protein affinity to DNA by in vitro transcription and RNA sequencing) are being used to build comprehensive TF binding atlases. DAP‐seq has already mapped thousands of TF binding profiles across multiple species [66, 67, 68, 69, 70], while PADIT‐seq detects even weak‐affinity binding events that often underlie crosstalk [71]. Applying these tools in tractable bacterial systems will help clarify the sequence and structural determinants that enable or constrain rewiring.

## 6 Conclusion

Looking forward, addressing these questions within our tractable experimental system aligns with several grand challenges in the field of regulatory evolution. Chief among these are: understanding how new regulatory functions arise without disrupting existing ones; identifying which TFs are most likely to be innovated under certain conditions, and why; quantifying the costs and benefits of crosstalk across ecological contexts; and predicting rewiring potential from first principles such as sequence, structure, or network context. By combining experimental evolution, high‐resolution transcriptomics, and predictive modelling, we aim to bridge mechanistic and theoretical approaches to GRN evolution, contributing not only to our understanding of how networks adapt, but also to our capacity to anticipate and shape that adaptation.

Gene regulation is often presented as deterministic: each TF activates only its cognate targets. But our work [9, 10, 11, 12] and others [72, 73, 74, 75, 76] show this view is incomplete. Crosstalk creates latent interactions that selection can amplify when beneficial. A regulator with slight promiscuity effectively explores additional regulatory space, generating phenotypic diversity within a clonal population without genetic change [77]. If environmental shifts render one of these interactions beneficial, selection can favor mutations that stabilize and strengthen it [52]. This perspective reframes the question from “why is crosstalk tolerated?” to “how does crosstalk shape evolvability?” Understanding the balance between the costs of misregulation and the benefits of enhanced flexibility remains a central challenge [78].

A future ambition is to translate these findings from controlled laboratory systems into both applied and natural contexts. In synthetic biology and biotechnology, principles of crosstalk and regulatory flexibility could guide the design of microbes with greater adaptive capacity [76]. For example, strains engineered to rewire in response to stress in industrial settings, or to exploit latent regulatory connections for novel metabolic outputs under certain environmental cues [79]. In ecological and clinical settings, the same principles help explain how natural populations of bacteria adapt so rapidly, for example, during a shift to a novel host environment [80, 81, 82, 83, 84] or early colonizers of the gut microbiome [85]. By understanding how rewiring plays out in nature, we can begin to define how and when crosstalk drives innovation, and ultimately how it shapes the evolvability of regulatory networks across life.

 

From our findings, we can derive general principles that shape GRN rewiring in bacteria, and possibly in other organisms:

- **Path of Least Resistance**: When regulatory function is lost, or a new one becomes beneficial, the evolutionary outcome is biased toward the pathway that requires the fewest and most probable mutational steps. Among the many theoretical ways a network could be rewired, the solution most easily reached through single or common mutations is the one most likely to occur, even if alternative solutions might offer comparable fitness benefits. This probabilistic bias contributes to the repeatability of evolutionary change, which may help predict which ones are accessible in practice.
- **Key Properties of Evolvable TFs**: High expression, high activation potential, and preexisting affinity to target genes are hallmarks of a rewirable TF. Such properties could be used to scan genomes and identify regulators with high evolutionary potential. For instance, global regulators or those with strong promoters and multiple input signals are statistically more likely to generate novel phenotypes than narrowly expressed, tightly insulated regulators. The broad, low‐specificity binding of the original TF provided latent affinities that mutations could quickly refine into new connections.
- **Modularity and Compatibility**: GRNs are modular in nature, due to expansion via duplication and specialization. Such modularity ‐ where an entire block of genes is controlled by a single input ‐ increases the likelihood that simple mutations can reassign control of the module. Many bacteria organize genes into operons or regulons that may be reattached to different regulators over evolutionary time. In higher organisms, developmental gene batteries can be redeployed under new regulators, similarly promoting evolutionary novelty in form and function.
- **Role of Crosstalk**: Low‐level, non‐cognate interactions mean that some alternative regulatory connections are already present at background levels, with negligible fitness effect. These interactions increase the probability that, should this interaction become beneficial, few mutational changes are needed to strengthen the interaction, repurposing a latent interaction into a functional connection. “Optimal crosstalk” represents a trade‐off between robustness and evolvability, while a perfectly specific network minimizes costly misregulation under stable conditions. Networks that tolerate some crosstalk can more readily adapt to change.

## Funding

T.B.T. is supported by the Royal Society via a Dorothy Hodgkin Research Fellowship (Grant no: DHF\R\231005 )

## Conflicts of Interest

The authors declare no conflict of interest.
